# TDLAS-Based Rapid and Accurate Measurement of Near-Ambient Temperature Using Near-Infrared Vibrational Water Vapor Transitions

**DOI:** 10.3390/s25092839

**Published:** 2025-04-30

**Authors:** Jiaao Zhang, Jiao Gao

**Affiliations:** Shien-Ming Wu School of Intelligent Engineering, South China University of Technology, Guangzhou 511442, China; jiaao_zhang@189.cn

**Keywords:** temperature measurement, tunable diode laser absorption spectroscopy, water vibrational transition, near-ambient environment

## Abstract

Tunable diode laser absorption spectroscopy (TDLAS) of water vapor transitions has been used to effectively measure temperature under high temperature and pressure conditions. However, due to the weak variation in transmittance and low signal-to-noise ratio, applying the same technique to measure temperature in near-ambient environments is difficult. This study reports the rapid and accurate measurement of near-ambient temperature through monitoring water vapor transitions with a three-point measurement method based on TDLAS. The transmission spectra of two selected water vibrational transitions at 1389.01 and 1389.89 nm are investigated, and the monotonic variations in the dip area are validated both theoretically and experimentally. The results show that by using the proper regression parameter (Ratio_dipA_/Ratio_dipB_)^2^, the temperature measurement time can be reduced to 40 s, with an uncertainty as low as 0.39 °C and a *p*-value as small as 1.98 × 10^−13^. This work contributes to rapid and accurate non-invasive temperature measurement in near-ambient complex environments.

## 1. Introduction

Rapid and accurate temperature measurement is essential in near-ambient environments (typically below 100 °C) [1,2,3,4], such as the separation of multicomponent biological particles, which requires temperature gradient control [5,6,7], and the analysis of natural convective flow, for which both liquid and vapor temperature need to be monitored simultaneously [8]. Conventional thermocouple measurement is commonly used because of its high accuracy, wide temperature range, and cost effectiveness. However, it exhibits inherent limitations in complex environments due to the invasive measurement requirement, contact-restricted single-point sampling, and intrinsic self-heating [9]. To date, various non-invasive thermometric methods have been proposed for complex thermal monitoring environments. The acoustic approaches determine temperature by analyzing thermally induced sound velocity variations through deploying multiple acoustic sources and receivers [10,11]. The optic approaches, such as Rayleigh scattering [12,13], coherent anti-Stokes Raman spectroscopy [14,15], and tunable diode laser absorption spectroscopy (TDLAS) [16], achieve high resolution measurement via characteristic light–matter interactions while requiring configured light sources and detectors.

Among them, TDLAS derives temperature through the quantitative analysis of the molecular absorption ratio at specific spectral transitions [17,18,19,20,21]. This method offers the advantages of simple implementation, cost efficiency, and wide spectral coverage, enabling its broad application in high-temperature (above 1000 °C) and high-pressure environments [22]. Zhang G. et al. employed TDLAS to quantitatively measure distributions of temperature and CO_2_ concentration in methane flames, demonstrating superior accuracy compared to thermocouple measurements [23]. Zhang T. et al. developed a TDLAS system for the simultaneous measurement of ethylene concentration and temperature (300–900 K) in combustion, demonstrating high stability for high-temperature environments [24]. There are several substances that can be used in TDLAS, and water vapor, which possesses intense near-infrared absorption transitions (1300–1600 nm), is a preferred target due to its distinct temperature-dependent spectral responses and the availability of low-cost commercial E-band lasers [25,26,27]. Liu et al. utilized water vapor absorption spectra to simultaneously measure temperature and diagnose operational states in internal combustion engines [28]. Rieker et al. integrated an in-cylinder TDLAS sensor into the spark plug, which enabled the real-time measurement of temperature and H_2_O concentration [29]. Ngo et al. exploited the pressure-dependent broadening of water absorption lines at ambient temperatures, developing a TDLAS-based non-contact pressure sensing system [30]. TDLAS with water vapor transitions has great potential for temperature measurement; however, compared to high temperature and pressure conditions, the weak absorbance of water vapor transitions under near-ambient conditions amplifies susceptibility to environmental disturbances. The resultant degradation in the signal-to-noise ratio compromises the accuracy of temperature measurement, demonstrating the need for further in-depth investigation.

In this study, TDLAS was performed with a single E-band tunable diode laser, focusing on two near-infrared water vapor transitions at 1389.01 nm (7199.4 cm^−1^) and 1389.89 nm (7194.8 cm^−1^) for temperature measurement in the near-ambient environment (23.5 °C to 53.1 °C at 1 atm). Both theoretical and experimental investigations of transmittance variations were conducted, quantifying the spectral dip areas of two water vapor transitions to validate the monotonic variation trends. To improve the accuracy and reduce the measurement time, a three-point measurement method was proposed. The transmittance ratios between experimental measurements and reference values at ambient temperature (23.5 °C) were quantitatively resolved, followed by a regression analysis to estimate the variation in temperature. Through systematic evaluation of multiple regression parameters, the measurement accuracy was further enhanced. The results demonstrate that in the near-ambient environment, the TDLAS-based three-point measurement method achieves temperature estimation within 40 s with an accuracy of 0.39 °C by utilizing the squared ratio of spectral dip parameter (Ratio_dipA_/Ratio_dipB_)^2^. This accuracy is lower than that of K-type thermocouples processing an accuracy of 0.42 °C [31]. The proposed three-point measurement method enables rapid, accurate, and non-invasive temperature estimation, expanding the applications of TDLAS to more complex thermal monitoring environments.

## 2. Experimental Apparatus and Methods

### 2.1. Fundamental Spectroscopy

Direct transmission spectroscopy for monochromatic radiation is governed by the Beer–Lambert law [32], in which the transmission *τ*(*σ*) at wave number *σ* can be defined as follows:

(1) $$\tau \left(\sigma \right)=\left(\frac{I_{t}}{I_{0}}\right)=\exp\left(-\alpha \left(\sigma \right)\right)\;,$$

where *I_t_* is the transmitted intensity, *I*_0_ is the incident intensity, and *α*(*σ*) is the spectral absorbance. For an optical path of length *L*, with a homogeneous mixture containing absorbing species with a mole fraction *x_a_* at constant total pressure *P* and temperature *T*, *α*(*σ*) is given by the following:

(2) $$\alpha \left(\sigma \right)=P\times L\times x_{a}\times \sum_{l}\varphi_{l}\left(\sigma ,\;P,\;T,x_{a}\right)S_{l}\left(T\right)\;,$$

where the sum extends over all absorbing lines *ℓ* of the species. *S_ℓ_*(*T*) and *φ_ℓ_*(*σ*, *P*, *T*, *x_a_*) are the integrated intensity and area normalized spectral shape of line *ℓ*, respectively. For integrated intensity *S_ℓ_*(*T*), it can be given by the following expression:

(3) $$S_{l}\left(T\right)=\frac{10^{-6}P_{0}}{k_{B}T}S_{l}^{0}\left(T_{0}\right)\frac{Q\left(T_{0}\right)}{Q\left(T\right)}\frac{1-e^{-\frac{hc\sigma_{l}}{k_{B}T}}}{1-e^{-\frac{hc\sigma_{l}}{k_{B}T_{0}}}}\frac{e^{-\frac{hcE_{l}}{k_{B}T}}}{e^{-\frac{hcE_{l}}{k_{B}T_{0}}}}\;,$$

where *T*_0_ is the reference temperature and $S_{l}^{0}(T_{0})$ is the integrated intensity at the reference temperature. The first term with *P*_0_ = 1.01 × 10^5^ Pa is a conversion factor. *Q*(*T*) is the rovibrational partition function [33], and *E_ℓ_* is the energy of the lower state of line *ℓ*. *h*, *c*, and *k_B_* are the Planck constant, the speed of light, and the Boltzmann constant, respectively. The spectral shape *φ_ℓ_*(*σ*, *P*, *T*, *x_a_*) represents the variation in the absorbance of a particular absorption transition, which reflects the perturbation of the transition energy level and the interaction between the absorbing species molecules and the laser beam. It is given by the following:

(4) $$\varphi_{l}\left(\sigma ,\;P,\;T,\;x_{a}\right)=\frac{1}{\pi }\frac{P\gamma_{l}(x_{a},\;T)}{{\left(\sigma -\sigma_{l}\right)}^{2}+{\left[P\gamma_{l}(x_{a},\;T)\right]}^{2}}\;,$$

where *σ_ℓ_* and *Pγ_ℓ_*(*x_a_*, *T*) are the position and pressure-broadened half-width at half maximum of the considered transition, respectively. In the case where the absorbing species is highly diluted in air, the pressure-broadening coefficient $\gamma_{l}(x_{a},\;T)$ is then simplified by $\gamma_{l}\left(x_{a},\;T\right)=\gamma_{l}^{air}(T)$. With the parameters $\gamma_{l}^{air}(T_{0})$ for the reference temperature and *n_ℓ_*, $\gamma_{l}^{air}(T)$ can be computed using the following:

(5) $$\gamma_{l}^{air}\left(T\right)=\gamma_{l}^{air}(T_{0}){\left(\frac{T_{0}}{T}\right)}^{n_{l}}\;\;.$$

Note that the spectral shape of line *ℓ* in Equation (5) is normalized to the unit area by integration over the wave number:

(6) $$\int_{-\infty }^{+\infty }\varphi_{l}\left(\sigma ,\;P,\;T,x_{a}\right)d\sigma =1\;,$$

so that for an isolated transition [34], the total area under the spectral curve can be calculated as follows:

(7) $$A=\int_{-\infty }^{+\infty }\alpha \left(\sigma \right)d\sigma =P\times L\times x_{a}\times \sum_{l}S_{l}(T)\;,$$

where *A* is the integrated absorbance.

### 2.2. Experimental Details and Data Analysis

**Figure 1:**
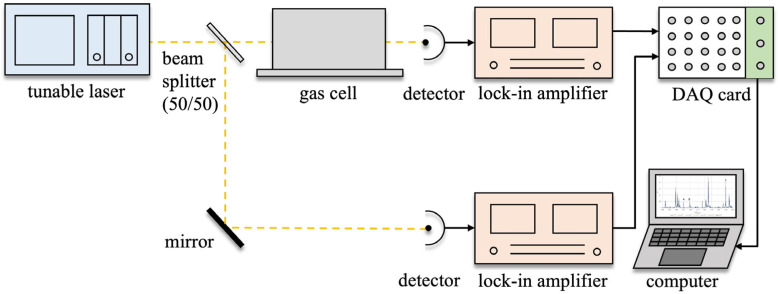
Schematic diagram of TDLAS configuration used for near-ambient temperature measurement.

The experimental setup used to perform TDLAS in the near-ambient environment is schematically shown in Figure 1. A tunable diode laser (Agilent (Santa Clara, CA, USA), 8163A) with an output power of 50 μW was selected. The laser beam was split into two beams, a measurement beam that was passed through a 10 cm long gas cell kept at the desired temperature and atmospheric pressure and a reference beam that traveled the same distance as the measurement beam but outside the gas cell. This allows the influence of baseline fluctuation and disturbances in the environment to be removed from the measurement. The transmitted laser beams were then detected by two separate InGaAs photodetectors (Thorlabs (Newton, NJ, USA), PDA20CS2). Because the intensities of the beams are weak and the variations in the water transmission dips are small for near-ambient environment measurements, the laser intensity was modulated at 1 kHz, and the transmission signals were recorded using a lock-in amplifier (NF Electronic Instruments (Yokohama, Japan), LI5640Y) connected to a data acquisition card (National Instrument (Austin, TX, USA), USB-6259). This measurement technique makes the measured signal insensitive to intensity variations of the laser source and thus increases the signal-to-noise ratio and improves the measurement accuracy [35,36]. The measured signals were recorded and processed on a personal computer.

Though water has numerous transitions in the near-infrared range (1300–1600 nm) in its 2*υ*_1_, 2*υ*_3_ and *υ*_1_ + *υ*_3_ vibration modes [37,38], the high-resolution transmission molecular absorption (HITRAN) simulation [39,40] was performed, and the variation in each dip as well as the related spectroscopic parameters were verified. The pressure was set to 1 atm, and the path length was fixed to 10 cm during the simulation. To measure the temperature in the near-ambient environment, two specific transitions at 1389.01 and 1389.89 nm were selected. These υ_1_ + υ_3_ band transitions exhibit strong absorption, with HITRAN simulations revealing contrasting temperature sensitivities due to their differing lower-state energies (*E_ℓ_* = 888.6 cm^−1^ for 1389.01 nm and 95.2 cm^−1^ for 1389.89 nm). Higher *E_ℓ_* transitions are weakened with the increase in temperature, while lower *E_ℓ_* transitions are strengthened, enabling enhanced temperature sensitivity through intensity ratio analysis. Additionally, they are spectrally isolated from neighboring dips, minimizing interference, and the integrated intensities ($S_{l}^{0}(T_{0})$ = 3.07 × 10^−21^ and 8.96 × 10^−22^) ensure a sufficient signal-to-noise ratio for a reliable near-ambient environment. During the TDLAS test, the desired temperature of the gas cell was controlled by a heater controller (Pike Technologies (Fitchburg, WI, USA), 6125), and the laser was then swept from 1388.91 to 1389.11 nm and from 1389.79 to 1389.99 nm. The step size of the sweep was 0.005 nm, with a dwelling time of 20 s between each step. The acquisition rate of the DAQ card was 300 Hz, and the total measurement time was 13 min for each dip. In general, the line shape of the target transition is usually fitted by the Voigt profile [41], but the Lorentzian profile [42] was selected to calculate the dip area of the water spectrum, as the Doppler width was much smaller than that of the pressure broadened one in the considered near-ambient environment.

In order to improve the accuracy and reduce the measurement time of the temperature measurement, a three-point measurement method that collected only the left edge, right edge, and minimum of the transmittance dip was attempted. During the test, the step size of the sweep was changed to 0.1 nm while all other parameters remained unchanged. It was possible to reduce the measurement time to 40 s for each dip. After baseline correction, the ratio of the dip intensity between the measurement and the reference laser beam was calculated as follows:

(8) $${Ratio}_{dip}=\frac{\left(\frac{I_{left}+I_{right}}{2}-I_{min}\right)}{\left(\frac{I_{left}^{\prime }+I_{right}^{\prime }}{2}-I_{min}^{\prime }\right)}\;,$$

where *I* and *I′* are the intensities of the measurement beam and the reference beam, respectively. The left, right, and min subscripts correspond to the wavelengths of 1388.91, 1389.11, and 1389.01 nm in the first sweep and 1389.79, 1389.99, and 1389.89 nm in the second sweep, respectively.

## 3. Experimental Results and Discussion

**Figure 2:**
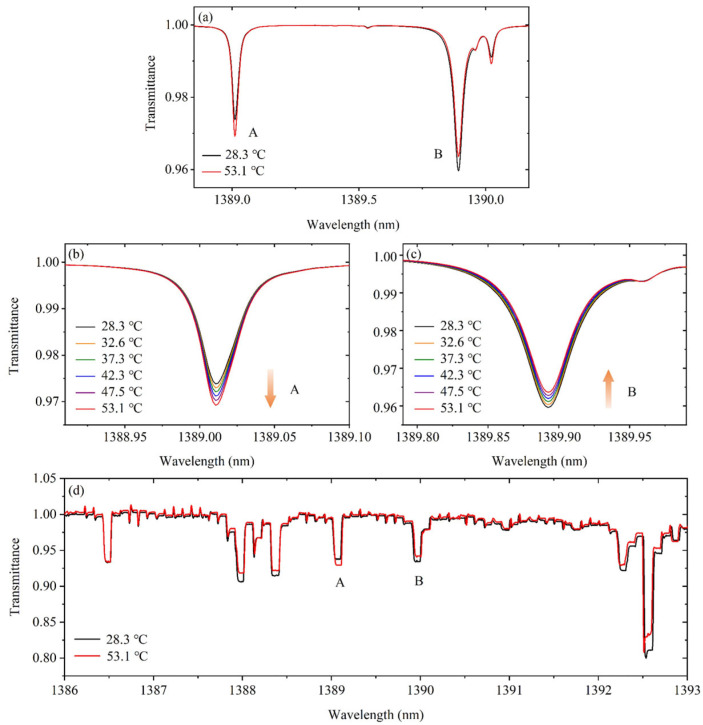
(**a**) Transmittance variations in 1389.01 nm (dip A) and 1389.89 nm (dip B) transitions of water vapor at 28.3 °C and 53.1 °C in HITRAN simulation. Detailed simulated transmittance variations in (**b**) 1389.01 nm and (**c**) 1389.89 nm transitions at different temperatures. (**d**) Experimental transmission spectra of two target transitions and their neighboring dips measured through TDLAS at 28.3 °C and 53.1 °C.

Figure 2a–c exhibit the HITRAN simulated variations in the transmission spectra of two water vapor transitions (1389.01 and 1389.89 nm) at different temperatures. It can be noticed that the transmittance changes systematically with temperature in the near-ambient environment. For dip A, the transmittance decreases as the temperature increases, whereas for dip B, the transmittance increases as the temperature increases. The spectroscopic parameters and the rovibrational partition function for selected water transitions are listed as shown in Table 1 and Table 2. The two selected dips both correspond to *υ*_1_ + *υ*_3_ vibration mode and at the reference temperature, the integrated intensities $S_{l}^{0}(T_{0})$ are 3.07 × 10^−21^ and 8.96 × 10^−22^ as well as the pressure-broadening coefficients $\gamma_{l}^{air}(T_{0})$ are 0.10 and 0.06, respectively. The relationship between the *Q*(*T*)/*Q*(*T*_0_) and *T*/*T*_0_ were evaluated, and the variation trend suggests that the temperature can be estimated from these two water vapor transitions at the desired temperature.

Through TDLAS, the two transmission spectra were experimentally collected as shown in Figure 2d. It is obvious that as temperature increases, the transmittance of dip A and B decreases and increases, respectively, showing the same variation trend as the simulated results. Moreover, within the range from 1386 to 1393 nm, the dip at 1386.48 nm shows no variation with the changing temperature. Multiple dips appear near 1388.13 nm and 1392.54 nm, which are accompanied by significant internal interactions. Compared to these dips, dip A and dip B are more sensitive and less related, indicating that they are of great potential to be used for temperature estimation in the near-ambient environment.

**Figure 3:**
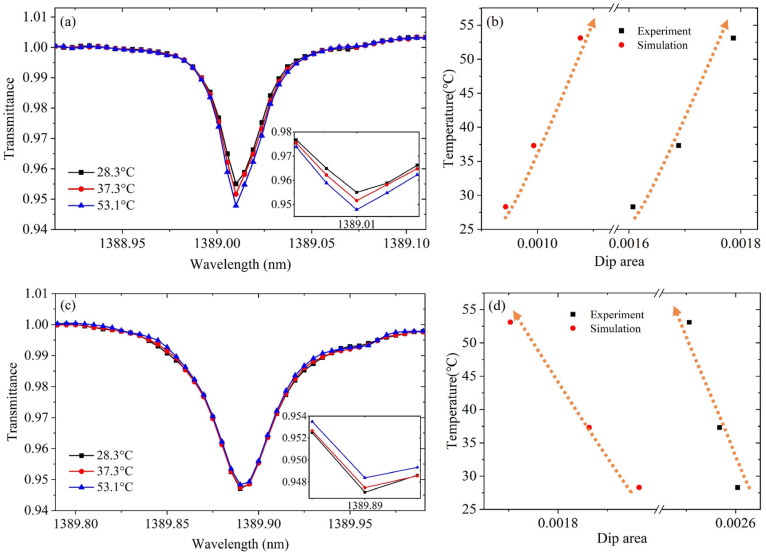
(**a**) Experimental transmission spectra of 1389.01 nm transition of water vapor at 28.3 °C, 37.3 °C, and 53.1 °C. (**b**) Dip areas of experimental and simulated spectra at each temperature. (**c**) Experimental transmission spectra of 1389.89 nm transition of water vapor at 28.3 °C, 37.3 °C, and 53.1 °C. (**d**) Dip areas of experimental and simulated spectra at each temperature. The insets are enlargements showing the transmittance variations around each dip. The dotted lines exhibit the variation trend of the dip areas.

For the selected transitions at 1389.01 and 1389.89 nm, the experimental transmission spectra at different temperatures are shown in Figure 3a,c. For the dip at 1389.01 nm, noticeably, as temperature increases from 28.3 °C to 53.1 °C, the transmission dip decreases proportionally, and no fluctuation in the baseline and the line shape is observed. As shown in Figure 3b, the dip areas of each experimental spectrum are 0.0016, 0.0017, and 0.0018, while the corresponding simulated spectral values are 0.0009, 0.0010, and 0.0011, respectively. It is obvious that compared to the simulated dip, the area values of experimental dip are slightly larger at each temperature. Moreover, as shown by the dotted line, the same monotonic increasing trend of experimental dip area is observed with the increase of temperature in the near-ambient environment.

Similarly, for the dip at 1389.89 nm, the experimental transmission spectra exhibit the same monotonic variation, i.e., as the temperature increases from 28.3 °C to 53.1 °C, the transmission dip increases. The baseline and the line shape remain unchanged, but compared to the above dip, the transmittance variation becomes smaller, indicating a lower sensitivity of this dip to the temperature changes. As shown in Figure 3d, the dip areas of each experimental and simulated spectrum are 0.0026, 0.00257, and 0.0025 as well as 0.0020, 0.0019, and 0.0017, respectively. It can be noticed that the area values of experimental dip are still slightly larger than those of simulated dip at each temperature. Moreover, the changing rate of the experimental dip area is slightly smaller than that of the simulated one, although the dotted line confirms the similar monotonically decreasing relationship between the area and the near-ambient temperature variation.

**Figure 4:**
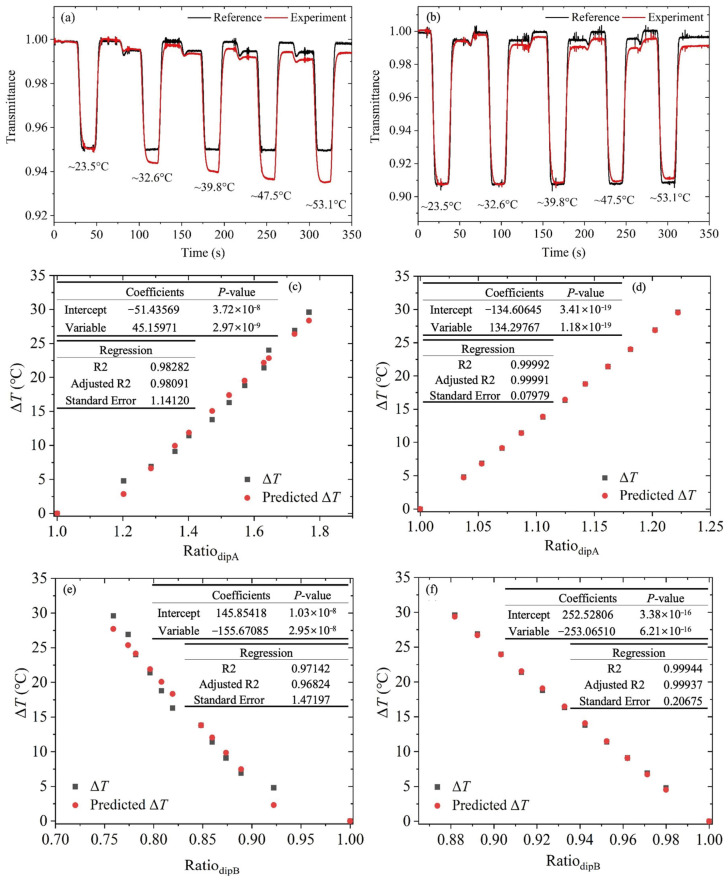
Transmittance of (**a**) 1389.01 nm and (**b**) 1389.89 nm transitions measured by the three-point measurement method at each desired near-ambient temperature. Regression analysis by using the (**c**) measured and (**d**) simulated Ratio_dipA_. Regression analysis by using the (**e**) measured and (**f**) simulated Ratio_dipB_.

To accurately and rapidly measure temperature in the near-ambient environment, a three-point measurement method was performed. Figure 4a shows the transmittance variation of the 1389.01 nm transition that is obtained by TDLAS as temperature increases from 23.5 °C to 53.1 °C. The three-point measurement was performed at each desired temperature, and it can be noticed that as temperature increases, the experimentally measured dip intensity decreases, meaning that the transmittance decreases. The regression analysis of the calculated Ratio_dipA_ and the temperature difference between the measurement and the reference beam Δ*T* is shown in Figure 4c. The results show that using only Ratio_dipA_ as the parameter, an estimation of the near-ambient temperature with an uncertainty of 1.14 °C and an extremely small probability value (*p*-value) of 2.97 × 10^−9^ can be obtained. To verify the estimation, the Ratio_dipA_ analysis for the simulated data was also performed, and as shown in Figure 4d, the same relationship exists with temperature, i.e., when Ratio_dipA_ increases, the Δ*T* increases is confirmed. The uncertainty of the temperature estimation from the simulated transition is 0.08 °C and the *p*-value is 1.18 × 10^−19^.

Figure 4b exhibits the variation in transmittance of the 1389.89 nm transition when the temperature is varied within the desired near-ambient range. It can be found that as temperature increases, the experimental dip intensity increases, indicating a transmittance increase. The regression analysis was conducted as shown in Figure 4e, and the results show that the uncertainty of the temperature estimation is 1.47 °C using only the calculated Ratio_dipB_, and the *p*-value is as small as 2.95 × 10^−8^. Compared to the dip at 1389.01 nm, the estimation of temperature for this dip is a little lower, which is consistent with the above in that it presents slightly lower sensitivity in the temperature measurement. The simulated Ratio_dipB_ analysis was also performed as shown in Figure 4f, and the same relationship of increasing Ratio_dipB_ and decreasing Δ*T* was obtained. The uncertainty of the temperature estimation is 0.21 °C with the *p*-value of 6.21 × 10^−16^. Noticeably, whether using Ratio_dipA_ or Ratio_dipB_, there is a difference in the temperature estimation between the experimental and simulated data, which may be because the 100% transmission correction is imperfect, especially for the measured transmission at the elevated temperature. The close agreement between R2 and adjusted R2 values in Figure 4c–f, with a minimal discrepancy (well below the 0.05 threshold), demonstrates that the model maintains statistical robustness against overfitting.

Compared to sweeping the whole water vapor transition and then calculating the Ratio_dip_, the temperature can be more rapidly and accurately measured by using the three-point measurement method. In the near-ambient environment, the temperature change is small, and the transmittance variation is weak, resulting in a low signal-to-noise ratio. As a result, the longer the measurement takes, the more susceptible the results are to the environmental changes, and the lower the accuracy of the estimation. The three-point measurement method can reduce the measurement time by 95%, and with the use of lock-in detection as well as the reference beam, the influence of the baseline and line shape variations on the transmittance can be removed. Therefore, regardless of whether Ratio_dipA_ or Ratio_dipB_ is used, a regression with low uncertainty and small *p*-value can be obtained, indicating that the three-point measurement method is highly effective for rapid estimation and provides more accurate temperature measurement in the near-ambient environment.

Despite the low uncertainties and statistically significant *p*-values obtained in the regression analysis employing Ratio_dipA_ and Ratio_dipB_, we investigated additional regression parameters to optimize the accuracy of temperature measurements under near-ambient conditions. As shown in Table 3, for one-parameter regressions, it can be noticed that the RA^2^ decreases the uncertainty, while the RB^2^ increases the uncertainty. Moreover, the RA/RB reduces the uncertainty, whereas its reciprocal increases the uncertainty. Therefore, by considering (RA/RB)^2^, a remarkably small uncertainty of 0.39 °C and an extremely low *p*-value of 1.98 × 10^−13^ are obtained, confirming that the regression parameter is highly reliable, and the near-ambient temperature can be more accurately estimated. Two-parameter regression is also analyzed and noticeably, all combinations of the RA and RB, RA and RA^2^, RB and RB^2^ contribute to reducing the uncertainty of the temperature measurement, thus by using the RA/RB and (RA/RB)^2^, the minimum uncertainty of 0.41 °C is obtained. The *p*-values of each parameter are 1.59 × 10^−3^ and 7.38 × 10^−1^. The R2 and adjusted R2 are 0.99797 and 0.99774, respectively. The small discrepancy confirms the strong generalization capability and robustness against overfitting of the model.

**Figure 5:**
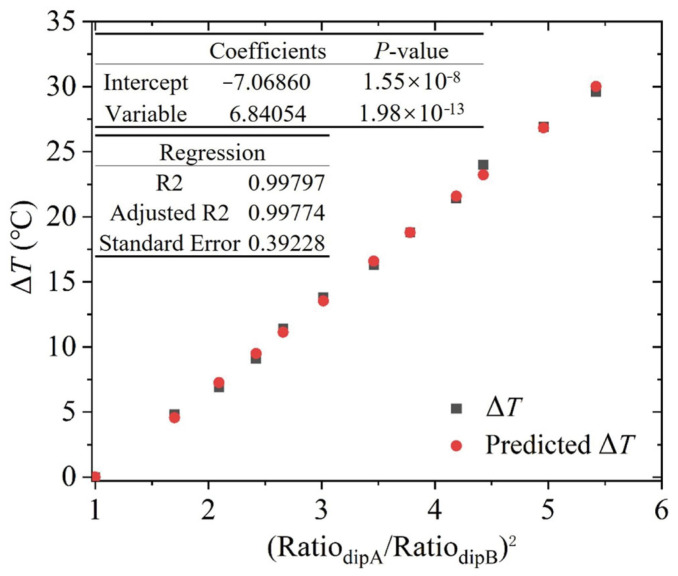
Results of the regression analysis using one parameter (Ratio_dipA_/Ratio_dipB_)^2^.

The regression analysis with (RA/RB)^2^ as the only parameter was plotted as shown in Figure 5. It can be noticed that there is almost no difference between the measured and the estimated Δ*T*, indicating that the temperature in the near-ambient environment is accurately estimated with the appropriate regression parameter by the TDLAS technique using the three-point measurement method.

## 4. Conclusions

In summary, tunable diode laser absorption spectroscopy was used to measure the temperature in the near-ambient environment. Two vibrational transitions of water vapor at 1389.01 and 1389.89 nm were selected for their high sensitivities and good isolation from other gas transitions. The HITRAN simulation was performed to validate the transmittance variation as temperature changes from 28.3 °C to 53.1 °C. The dip areas of each experimental and simulated spectrum were calculated, and the results show that similar to the simulation, the monotonic variations in dip area of two water transitions are confirmed. To accurately and rapidly measure the near-ambient temperature, a three-point measurement method was performed, which can reduce the measurement time by 95%. The regression analysis of the calculated Ratio_dip_ and the temperature difference Δ*T* was analyzed. The results show that by using only Ratio_dipA_ or Ratio_dipB_, temperature estimations with uncertainties of 1.14 °C or 1.47 °C are obtained, and the corresponding *p*-values are as small as 2.97 × 10^−9^ or 2.95 × 10^−8^, indicating that the three-point measurement method is highly effective and the temperature can be reliably measured in the near-ambient environment. Furthermore, different regression parameters are investigated to continually improve the accuracy of the temperature measurement, and the uncertainty of 0.39 °C as well as the *p*-value of 1.98 × 10^−13^ are obtained using the regression parameter (Ratio_dipA_/Ratio_dipB_)^2^. The small uncertainty and the *p*-value demonstrate that the near-ambient temperature can be rapidly and accurately estimated with the proper regression parameter by the TDLAS technique using the three-point measurement method. This work promises a rapid and accurate technique for non-invasive temperature measurement in near-ambient complex environments.

## Figures and Tables

**Table 1:** Spectroscopic parameters for selected water transitions based on HITRAN simulation.

$\sigma_{l}$ ^1^ (cm^−1^)	*λ* (nm)	Vibration Modes	$n_{l}$	$E_{l}$ (cm^−1^)	$S_{l}^{0}(T_{0})$ ^2^ (cm^−2^/(molec/cm^3^))	$\gamma_{l}^{air}(T_{0})$ (cm^−1^/atm)
**7194.81**	1389.89		0.69	95.20	3.07 × 10^−21^	0.10
**7199.38**	1389.01		0.62	888.60	8.96 × 10^−22^	0.06

^1^ The pressure-induced spectral shift is disregarded. ^2^ *T*_0_ is the reference temperature, equivalent to 23 °C (296 K).

**Table 2:** Values of rovibrational partition function at different temperatures for selected water transitions based on HITRAN simulation.

*T* (°C)	*Q*(*T*)	*Q*(*T*)/*Q*(*T*_0_)	*T* (°C)	*Q*(*T*)	*Q*(*T*)/*Q*(*T*_0_)
**0**	154.72066	0.88599	5	159.00232	0.91051
**10**	163.32625	0.93527	15	167.69207	0.96027
**20**	172.09942	0.98551	25	176.54793	1.01098
**30**	181.03724	1.03669	35	185.56697	1.06263
**40**	190.13676	1.0888	45	194.74624	1.11519
**50**	199.39505	1.14181	55	204.08282	1.16866
**60**	208.80918	1.19572			

**Table 3:** Uncertainties and *p*-values of different parameters for the regression analyses.

		RA	RA^2^	RA, RA^2^	RB	RB^2^	RB, RB^2^
**Standard Error**		1.14120	0.75644	0.56913	1.47197	1.68444	0.69683
***p*-value**	P1	2.97 × 10^−9^	7.32 × 10^−11^	7.20 × 10^−4^	2.95 × 10^−8^	9.98 × 10^−8^	4.70 × 10^−4^
	P2	/	/	2.28 × 10^−2^	/	/	1.56 × 10^−4^
		RA, RB ^1^	RA/RB	RB/RA	(RA/RB)^2^	(RB/RA)^2^	RA/RB, (RA/RB)^2^
**Standard Error**		1.20965	0.75223	2.11287	0.39228	2.79582	0.41298
***p*-value**	P1	9.22 × 10^−1^	6.96 × 10^−11^	7.74 × 10^−7^	1.98 × 10^−13^	9.81 × 10^−6^	1.59 × 10^−3^
	P2	4.98 × 10^−2^	/	/	/	/	7.38 × 10^−1^

^1^ RA and RB are the abbreviations of Ratio_dipA_ and Ratio_dipB_, respectively.

## Data Availability

Data are contained within the article.
